# Epidemiologic Analysis of a Postelimination Measles Outbreak in Central Ohio, 2022–2023

**DOI:** 10.1001/jamanetworkopen.2024.29696

**Published:** 2024-08-01

**Authors:** Rosemary A. Martoma, Matthew Washam, Hinda Omar, Ava R. Martoma, Randal De Souza, Sagar Kumar, Robert D. Sege, Emily E. Ricotta, Maimuna S. Majumder

**Affiliations:** Department of Pediatrics, The Ohio State University College of Medicine, Columbus; Division of Primary Care Pediatrics, Nationwide Children’s Hospital, Columbus, Ohio; KidsMates Inc, Boca Raton, Florida; Florida Atlantic University Schmidt College of Medicine, Boca Raton; Department of Pediatrics, The Ohio State University College of Medicine, Columbus; Division of Infectious Diseases, Nationwide Children’s Hospital, Columbus, Ohio; Infectious Disease Epidemiology, Prevention and Control Division, Minnesota Department of Health, Saint Paul; KidsMates Inc, Boca Raton, Florida; Division of Infectious Diseases, Nationwide Children’s Hospital, Columbus, Ohio; Northeastern University, Boston, Massachusetts; Tufts Medical Center, Institute for Clinical Research and Health Policy Studies, Boston, Massachusetts; Epidemiology and Data Management Unit, Division of Intramural Research, National Institute of Allergy and Infectious Diseases, National Institutes of Health, Bethesda, Maryland; Computational Health Informatics Program, Boston Children’s Hospital, Boston, Massachusetts; Department of Pediatrics, Harvard Medical School, Boston, Massachusetts

## Abstract

**IMPORTANCE:**

Postelimination outbreaks threaten nearly a quarter century of measles elimination in the US. Understanding these dynamics is essential for maintaining the nation’s measles elimination status.

**OBJECTIVE:**

To examine the demographic characteristics and transmission dynamics of the 2022 to 2023 central Ohio measles outbreak.

**DESIGN, SETTING, AND PARTICIPANTS:**

This cross-sectional study used electronic medical records and publicly available measles reports within an extensive central Ohio primary care network involving inpatient and outpatient settings. Participants included 90 children in Ohio with confirmed measles cases in 2022.

**EXPOSURE:**

The exposure of interest was confirmed measles cases in Ohio in 2022. This included 5 internationally imported cases and 85 locally acquired cases.

**MAIN OUTCOMES AND MEASURES:**

The primary outcome involved documenting and analyzing confirmed measles cases in Ohio in 2022, focusing on demographic characteristics, immunization status, and transmission links in outbreak-related cases.

**RESULTS:**

This study analyzed 90 measles cases (47 [52.2%] male participants) in Ohio during 2022. Most participants self-identified as African or American Black (72 [80.0%]), with additional race categories including Asian, Hispanic, multirace (6 [6.7%]), White, and unknown (6 [6.7%]). Most participants were of Somali descent (64 [71.1%]), with additional ethnicity categories including American (16 [17.8%]), Guatemalan, Nepali, and unknown (6 [6.7%]). Participants were predominantly younger than 6 years (86 [95.5%]), unimmunized (89 [98.9%]), and resided in Franklin County, Ohio (83 [92.2%]). Prior to November 20, 2022, all cases occurred among unimmunized children of Somali descent in the Columbus area. Nosocomial superspreading events expanded the outbreak beyond the initially affected community.

**CONCLUSIONS AND RELEVANCE:**

This cross-sectional study of measles cases in Ohio during 2022 found that the outbreak primarily affected unimmunized children of Somali descent, highlighting the necessity for culturally tailored public health strategies to maintain measles elimination in the US. These findings underscore the importance of implementing targeted interventions and enhancing community engagement to increase vaccination rates.

## Introduction

Measles is a vaccine-preventable, potentially fatal, acute respiratory illness. The virus infects more than 90% of exposed, susceptible people.^1^ Airborne transmission of aerosolized droplets can persist for up to 2 hours in an enclosed space after an infectious person vacates.^1^ The prodromal period of infection is characterized by high fever, cough, congestion, and conjunctivitis. Koplik spots—distinct white lesions on the buccal mucosa—are pathognomonic for measles.^1^ A distinctive maculopapular rash emerges on the face at the hairline 3 to 5 days after the prodrome, extends downward, and radiates to the extremities.^1^ Individuals are considered infectious within 4 days of rash onset.^1^ Before the exanthem erupts, measles can resemble less harmful respiratory infections, which may lead to undetected transmission and delayed diagnosis.^2^

In 2000, the US qualified for measles elimination status after the continuous absence of endemic disease transmission for 12 months.^3^ The cornerstone of measles elimination is high population immunity achieved by routine measles, mumps, and rubella (MMR) immunization at 12 to 15 months and 4 to 6 years of age.^4^ A fully susceptible population requires a 2-dose MMR vaccination coverage of at least 93% to prevent outbreaks^2^; coverage below this threshold indicates the community is underimmunized, increasing the risk of disease spread. In measles-eliminated countries, the index case of an outbreak must be internationally imported. Adequate surveillance is an essential component of elimination. The Centers for Disease Control and Prevention (CDC) requires US health care professionals to report suspected measles cases to their designated health departments.^5^ Prompt investigation of cases and contacts is integral to containing disease transmission.

In 2014, the US experienced a 383-case postelimination outbreak after international measles importations from the Philippines led to 4 months of transmission in an underimmunized Amish community in Holmes County, Ohio.^6^ The Ohio Department of Health (ODH) and the CDC contained the outbreak with an effective rapid response centered around a successful immunization campaign.^6^ Most other large US postelimination outbreaks have occurred in close-knit, underimmunized communities.^3^ The eighth and most recent large US postelimination outbreak occurred in central Ohio in 2022. While many states require vaccination reporting, Ohio does not, rendering its state registry (Impact Statewide Immunization Information System) incomplete.^7^ However, a statistical model—VaxEstim, developed by Martoma et al^2^—estimated a 53% vaccination coverage in the exposed population at the onset of the 2022 to 2023 central Ohio measles outbreak.

On June 16, 2022, the ODH reported Ohio’s first case of internationally acquired measles since 2019.^8^ On November 5, an extensive central Ohio primary care network (PCN) reported 2 suspected cases of locally acquired measles to Columbus Public Health (CPH) and the ODH. Laboratory testing confirmed these cases.^9^ By November 9, 2022, CPH declared a local measles outbreak and began contact tracing 4 international importations, although the dates of these importations were not disclosed.^10^ During a November 30 press conference, CPH announced that, in collaboration with the CDC, they had linked the outbreak to a summer index case.^11^ An August 2023 CDC report later confirmed 85 locally acquired cases in central Ohio children between October 22 and December 24, 2022.^9^ The report also revealed that the 4 international measles cases were imported from East Africa to Franklin County and that a definitive index case was not established.^9^ The official end of the outbreak was declared on February 4, 2023, following 2 incubation periods without measles transmission. All tested cases shared the same B3 genotype circulating in East Africa.^9^ Finally, on February 3, 2024, the ODH confirmed 90 measles cases in Ohio during 2022.^12^ To our knowledge, this study presents the first detailed account of the 5 international importations, describes the distinctive demographic characteristics of the affected underimmunized community, and provides insights into the transmission dynamics of the 2022 to 2023 central Ohio measles outbreak.

## Methods

We designed a cross-sectional study of children with confirmed active measles disease (henceforth referred to as a case) in Ohio in 2022. The Nationwide Children’s Hospital Institutional Review Board approved this human participants’ research and waived the requirement for obtaining informed consent because this study posed minimal risk to participants and maintained their confidentiality. We followed the Strengthening the Reporting of Observational Studies in Epidemiology (STROBE) reporting guideline for cross-sectional studies.

### Data Sources

We obtained comprehensive epidemiologic and clinical data from individual electronic medical records (EMRs) for all PCN cases. The PCN routinely collects demographic data for all its patients to ensure thorough and responsive health care delivery to the diverse needs of its patient population. In our study, each patient’s guardian self-reported a single race, ethnicity, and language in the demographics section of the EMR. Race was assessed to identify and understand disparities in measles transmission dynamics and immunity gaps. Guardians selected 1 of the following 12 race categories: African; American Indian or Alaska Native; Asian; Black or African American; Latino Hispanic Black; Latino Hispanic unspecified; Latino Hispanic White; multirace; Native Hawaiian or Other Pacific Islander; White; guardian unavailable to ask; and patient or family declined. Guardians selected 1 language from 461 language categories; however, English, Nepali, Somali, and Spanish were the only languages chosen by the participants in this study. A list of 104 ethnicity selections is given in eAppendix 1 in Supplement 1. Notably, when a patient’s ethnicity was entered as American and the language as Somali, we categorized the patient’s ethnicity as Somali. This approach aligned with our understanding that language can reflect cultural affiliation more distinctly than nationality alone.

We located aggregate case numbers, sex, age, immunization status, and residential data from publicly available reports published by CPH on February 10, 2023,^13^ the CDC on August 4, 2023,^9^ and the ODH on February 3, 2024.^12^ We used an established measles serial interval of 6 to 29 days, defined as the time between symptom onset in 2 consecutive cases in a chain of transmission.^14^ A description of the laboratory confirmation of cases is detailed in eAppendix 2 in Supplement 1.

### Infectious Exposure and Epidemiologic Linking

We analyzed clinic encounter date and time stamps to assess clinic exposures, residential addresses to assess household exposures, and documentation of confirmed infectious sources to identify other (including daycare) exposures. We determined an infectious exposure had occurred in the clinical setting if a new case developed after a patient visited a clinic simultaneously or up to 2 hours following the departure of another patient with a confirmed case. We epidemiologically linked 2 cases if rash onset in the secondary case occurred within the 6- to 29-day measles serial interval of rash onset in the primary case to which it had an infectious exposure.

### Plausible Index Case

To identify a plausible index case from international importations, we used epidemiologic linking by subtracting the measles serial interval (6 to 29 days) from the rash onset date of the first locally acquired case. This calculation defined the time frame for initial exposure, pinpointing the likely index case.

### Case Classification

We designated cases as international importations if measles exposure occurred outside the US within the serial interval and all other cases as locally acquired. We determined that international importations were unrelated to the outbreak if we could not qualify them as a plausible index case or epidemiologically link them to a locally acquired case. We labeled the plausible index case as case 0, the first locally acquired case as case 1, and subsequent cases sequentially by date of rash onset. The date of rash onset determination is further described in eAppendix 3 in Supplement 1.

### Statistical Analysis

To summarize the demographic and clinical characteristics of the study participants, we calculated descriptive statistics using Stata, version 16 (Stata Corp). For spatial analysis, we used Google Maps to determine the distance between cases, and Stadia Maps (stadiamaps.com) and OpenStreetMap (openstreetmap.org/copyright) in R version 4.4.0 (R Project for Statistical Computing) to analyze the outbreak’s geographic distribution.

## Results

We identified 90 measles cases in Ohio between June 12 and December 24, 2022, including 47 (52.2%) male and 43 (47.8%) female participants. The cases were distributed as either African or African American or Black (72 [80.0%]), Asian, Hispanic, multirace (6 [6.7%]), White, and unknown (6 [6.7%]) race. Ethnically, the breakdown was American (16 [17.8%]), Guatemalan, Nepali, Somali (64 [71.1%]), and unknown (6 [6.7%]). The age distribution at rash onset was 25 children (27.8%) younger than 1 year, 24 children (26.7%) 1 year, 26 children (28.9%) 2 to 3 years, 12 children (13.3%) 4 to 6 years, and 3 children (3.3%) 9 to 14 years. Regarding immunization status, 25 participants (27.8%) were unimmunized and not yet eligible for routine immunization, 49 participants (54.4%) were unimmunized although eligible for partial 1-dose immunization, 15 participants (16.7%) were unimmunized although eligible for full immunization, and 1 participant (1.1%) was age-appropriately partially immunized. Geographically, 83 participants (92.2%) resided in Franklin County, 2 participants (2.2%) in Madison County, and 1 participant (1.1%) each in Clark, Fairfield, Richland, Ross, and Union counties. Before November 20, cases occurred exclusively in children of Somali descent residing in the Columbus area. All cases were localized within a 65-mile radius of Columbus (Figure).

We located 84 cases (93.3%) from the PCN EMRs, including 5 international importations and 79 locally acquired cases, and we obtained 6 locally acquired cases (6.7%) from public health reports with limited aggregate data. Our outbreak analysis excluded 4 unrelated international importations, leaving 86 cases, including 1 plausible index case on October 8, 2022, (case 0) and 85 locally acquired cases between October 22 and December 24 (cases 1 to 85). Additionally, our network diagram excluded 6 locally acquired cases (cases 80 to 85) with unknown rash onset dates, leaving 80 cases.

### International Importations

#### Before the Outbreak: June 12 to October 8, 2022

The first international importation of measles in Ohio occurred in an unimmunized female 17 months of age of Somali descent following a 2-month stay in Kenya. She experienced cough, congestion, conjunctivitis, and fever 2 days after returning to Columbus. Five days later, on June 12, she was admitted to the PCN hospital for dehydration. Physical examination revealed a faint maculopapular rash on her face and abdomen and Koplik spots on her buccal mucosa. The hospital discharged her after 2 days with instructions to quarantine at home pending test results. Laboratory tests confirmed measles infection 2 days later.

A second international measles importation occurred in an unimmunized male 29 months of age of Somali descent who returned to Columbus after traveling to Kenya for 2 months. Two days after his return, he developed a high fever, cough, congestion, conjunctivitis, and vomiting. On September 2, his mother called the triage line, concerned about her son’s symptoms after international travel to an area with endemic measles. The triage nurse instructed her to bring her child to the PCN for evaluation. Later that day, the patient arrived in the PCN Emergency Department and was diagnosed with COVID-19. After discharge, a rash emerged on his face, and his symptoms worsened. The following day, the hospital admitted him for dehydration. He tested positive for measles infection and *Salmonella enteritis* during a 6-day hospital stay.

A third international measles importation occurred in an unimmunized female 15 months of age of Somali descent who resided in Nairobi, Kenya, for a year. She developed a fever, cough, congestion, and vomiting 3 weeks before returning to Columbus. A week later, on September 14, a rash emerged. She presented to the PCN emergency department the day she returned but left without being seen. She was admitted to the hospital for dehydration the following day. Physical examination revealed a faint, erythematous, maculopapular rash on her cheeks and back and Koplik spots on her buccal mucosa. During her 2-day hospital stay, laboratory testing confirmed measles infection.

A fourth international measles importation occurred in an unimmunized male 3 years of age of Somali descent whose illness began with cough and congestion shortly before returning to Columbus from an 18-month stay in Kenya. Several days after returning, he developed a fever and vomiting. He was diagnosed with otitis media at a local clinic. However, since his symptoms did not resolve with antibiotic treatment, he presented to a PCN emergency department and a local clinic in the ensuing days. On October 8, he developed a diffuse, erythematous rash on his face, spreading to his chest and back. The hospital admitted him for an evaluation of his fever of unknown origin. During his 4-day admission, laboratory testing confirmed measles infection.

#### During the Outbreak: October 8 to December 24, 2022

A fifth international measles importation to Ohio occurred during the measles outbreak. An unimmunized male 14 months of age of Somali descent, residing in Kenya for 8 months, was admitted to a Kenyan hospital with an oxygen requirement secondary to pneumonia. A week after discharge, in late November, he developed a rash and was presumed to have measles. In early December, the day after returning to Columbus, he presented to the emergency department with a worsening cough and new-onset fever. He was diagnosed with influenza B and discharged with instructions to quarantine at home pending test results. Laboratory testing confirmed measles infection 2 days later. We did not epidemiologically link this case to any outbreak-related cases.

### Plausible Index Case (Case 0)

We calculated potential rash onset dates for a plausible index case by subtracting the 6- to 29-day measles serial interval from the first confirmed locally acquired case on October 22, yielding a range from September 23 to October 16. The October 8 international importation was the only case to meet this criterion; however, it did not qualify as a definitive index case due to the absence of direct infectious exposure.

### Locally Acquired Cases

We epidemiologically linked the first locally acquired case on October 22 (case 1) to 4 secondary infections, 3 in the household (cases 3, 5, and 13) and 1 in a PCN urgent care clinic (case 4). Case 4, the first identified hospitalization of a locally acquired case, was linked to 3 tertiary household infections (cases 16, 19, and 21). Cases 19 and 21 presented to a PCN urgent care clinic on November 7 and were epidemiologically linked to 10 quaternary nosocomial infections between November 20 and 26. Following a daycare exposure with unknown rash onset, case 17 presented to the same PCN urgent care clinic on November 7 and was linked to 11 quaternary nosocomial infections; of these infections, 10 were simultaneously exposed to cases 19 and 21. We associated nosocomial transmission with the dissemination of measles infection outside the community of children of Somali descent and later the Columbus area (eFigure in Supplement 1).

### Outbreak-Related Cases

Of the 86 outbreak-related PCN cases, we epidemiologically linked 60 (69.8%), including 1 index case (1.2%) imported from Kenya, 25 exposures (29.1%) at a single PCN urgent care clinic, 11 exposures (12.8%) at the PCN emergency department, 3 exposures (3.5%) at 3 other PCN locations, 4 exposures (4.7%) at daycare or school, 10 household exposures (11.6%) to infections acquired at the PCN, and 6 household exposures (7.0%) to infections of an unknown origin (eFigure in Supplement 1).

## Discussion

This cross-sectional study of measles cases in central Ohio during 2022 observed that the outbreak primarily affected unimmunized children of Somali descent. A total of 93.3% of all measles cases in Ohio during 2022 were tested and documented at a central Ohio PCN with comprehensive EMRs, offering a rare opportunity to extensively examine the epidemiology of a large US postelimination outbreak. Notably, a 2021 study of PCN EMR data showed a decline in 1-dose MMR vaccination among children 16 months of age, from 72% in 2017 to 2020 to 62% by mid-2020, signaling a growing immunity gap.^15^ While state registry data are incomplete, a report estimated that 88.3% of Ohio kindergarten children received the 2-dose MMR vaccination during the 2021–2022 school year.^16^ These data suggest local and state underimmunization in late 2022, setting the stage for a central Ohio outbreak with the potential for statewide spread.

International importations into close-knit, underimmunized communities characterize postelimination outbreaks.^3^ Our study demonstrates that, during the initial stage of the 2022 to 2023 central Ohio measles outbreak, endemic transmission occurred exclusively in unimmunized children of Somali descent in the Columbus area. Columbus, Ohio, has the second-largest Somali community in the US, following Minneapolis, Minnesota. Both communities actively share information through a rich tradition of oral communication.^17^ Following antivaccination campaigns targeting the Somali community with false assertions of an association between MMR and autism, vaccination rates in Minnesota-born children of Somali descent 2 years of age precipitously declined from 91% in 2004 to 54% in 2010.^18^ In 2011, a 21-case measles outbreak began in Hennepin County, Minnesota, after an unimmunized child 30 months of age of Somali descent returned from Kenya.^19^ The outbreak affected children with a median age of 12 months; none were age-appropriately vaccinated, and 38% were of Somali descent.^19^ Since 2011, the Minnesota Department of Health has actively engaged the Somali community in an attempt to increase MMR vaccination coverage.^18^ However, MMR vaccination rates dropped even further, to 42% in Minnesota and 36% in Hennepin County by 2017, when a 75-case postelimination outbreak affected children with a median age of 2 years; 91% were unvaccinated, and 81% were of Somali descent.^19^ Similar to their Minnesotan counterparts, members of the Columbus Somali community have cited concerns about autism as a reason for MMR refusal.^20,21^

Our study reveals that prior to the November 9, 2022, outbreak declaration, nosocomial transmission within the PCN had already precipitated superspreading events, extending the measles outbreak beyond the close-knit Somali community in Columbus. This underscores the swift risk escalation from multiple international importations in mid to late 2022. Following the official outbreak declaration, in partnership with the CDC and local health departments, the PCN implemented several successful rapid responses to halt locally acquired transmission. During this outbreak, off-site laboratory testing led to diagnostic delays, and pervasive vaccine hesitancy required alternate rapid responses to contain the spread of measles.^7^ These responses included a media awareness campaign, quarantines, contact tracing, daycare closures, laboratory testing of high-risk patients in negative flow rooms, and clinician education.

### Limitations

Our study has limitations, notably the reliance on self-reported data, which is susceptible to biases from misclassification or inaccuracies. The absence of race and ethnicity data for 6 individuals not assessed at the PCN potentially distorted the analysis of transmission patterns. Additionally, our insights into transmission dynamics were limited by self-reported data concerning rash onset dates and infectious exposures outside the PCN. These limitations underscore the need for cautious interpretation of our findings. Enhanced granularity in these data could significantly improve our understanding of transmission dynamics within these critical epidemiological contexts.

## Conclusions

This cross-sectional study describing a large measles infection outbreak in central Ohio during 2022 observed that the outbreak primarily affected unimmunized children of Somali descent and highlighted the critical need for vigilant monitoring of international importations and effective management of exposed individuals through enhanced collaboration between community leaders and health systems. Ultimately, understanding the origin of vaccine hesitancy and the beliefs of those who choose not to immunize their children will offer insights into closing immunity gaps and preventing future measles outbreaks. Integrating comprehensive measures such as developing targeted educational programs, strengthening community engagement, ensuring the availability of accurate information, addressing misinformation, and improving vaccine accessibility is essential. A unified and adaptive public health approach is pivotal for building trust and should involve community stakeholders and health professionals to ensure broader applicability and effectiveness across vaccine-preventable diseases.

## Supplementary Material

Supp online content

Supp 2

SUPPLEMENT 1.

**eAppendix 1.** Ethnicity Categories

**eAppendix 2.** Laboratory Confirmation of Measles Cases

**eAppendix 3.** Classification of Measles Cases by Date of Rash Onset

**eFigure.** Network Diagram of the 2022–2023 Central Ohio Measles Outbreak

eReference

SUPPLEMENT 2.

Data Sharing Statement

## Figures and Tables

**Figure.**
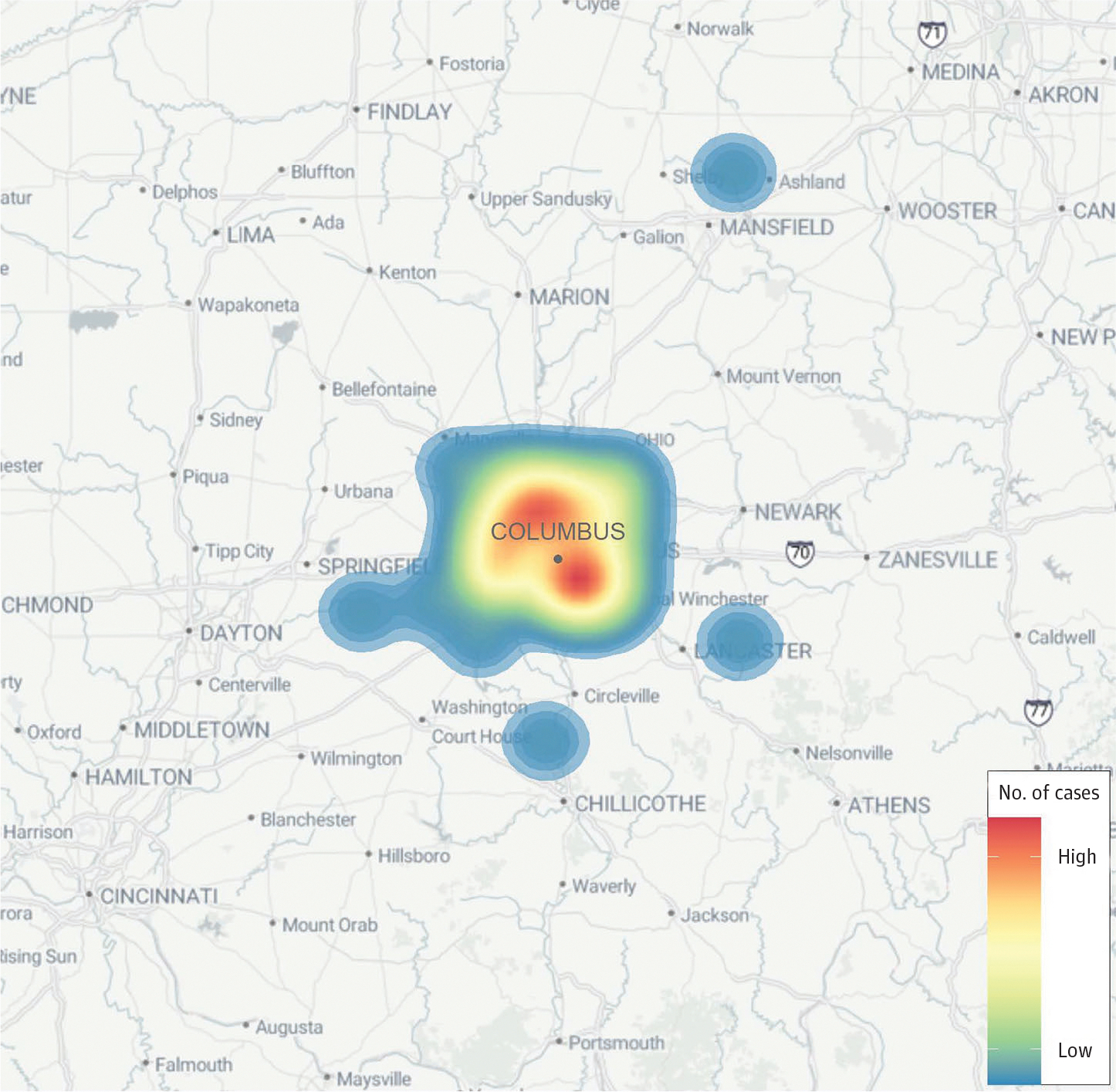
Geographical Distribution of the Central Ohio Measles Outbreak From 2022 to 2023 This figure depicts the residential locations of 86 measles infection cases identified as outbreak-related, with a predominance of cases in the Columbus, Ohio, area. The locations are approximate to protect the identities of the affected individuals.

## Data Availability

See Supplement 2.

## Key Points

### Question

What are the unique demographic characteristics and transmission patterns of the 2022 to 2023 central Ohio measles outbreak?

### Findings

This cross-sectional study analyzing 90 measles cases in Ohio from June 12 to December 24, 2022, found that before November 20, the outbreak exclusively affected unimmunized children who resided in the Columbus area and were identified as Somali. Nosocomial transmission extended the outbreak beyond this community.

### Meaning

This study highlights an association between a large US postelimination measles outbreak and a close-knit, vaccine-hesitant community, pinpoints health care settings as a significant transmission risk, and indicates a need for customized public health strategies to maintain US measles elimination.
