# Supplementary material for: Epidemiologic Analysis of a Postelimination Measles Outbreak in Central Ohio, 2022–2023
Source: JAMA Netw Open. Author manuscript; Available in PMC 2025 Oct 9. (PMC12509376; doi:10.1001/jamanetworkopen.2024.29696)
Supplement: Supp online content [file NIHMS2109843-supplement-Supp_online_content.pdf]

Supplementary Online Content

Martoma RA, Washam M, Hinda Omar H, et al. Epidemiologic Analysis of a Postelimination Measles Outbreak in Central Ohio, 2022-2023. *JAMA Netw Open*. 2024;7(8):e2429696. doi:10.1001/jamanetworkopen.2024.29696

eAppendix 1. Ethnicity Categories
eAppendix 2. Laboratory Confirmation of Measles Cases
eAppenidx 3. Classification of Measles Cases by Date of Rash Onset
eFigure. Network Diagram of the 2022-2023 Central Ohio Measles Outbreak
eReference.

This supplementary material has been provided by the authors to give readers additional information about their work.

© 2024 Martoma RA et al. *JAMA Network Open*.

eAppendix 1. Ethnicity Categories (104 categories)

Guardians selected one language from among the following 104 ethnicity categories:

Afghanistani/Afghan/Afghani
African, Alaskan, Albanian
American
Appalachian
Arab/Arabic
Asian Indian
Assyrian
Bahamian
Bangladeshi
Bantu
Bolivian
Brazilian
British/English
Bulgarian
Burmese
Cambodian
Cameroonian
Canadian
Cape Verdean
Caribbean Islander
Central African
Central African Republic
Central American
Chinese, Colombian
Costa Rican
Cuban
Dominican, Islander
Dominican
Dutch
Eastern African
Eastern European
Ecuadorian
Egyptian
Eritrean
Ethiopian
European
Filipino
French
Gambian
German
Ghanaian/ Ghanian
Greek
Guatemalan
Guinea
Haitian
Honduran
Hong Kong
Indonesian
Iranian
Iraqi
Irish
Israeli

© 2024 Martoma RA et al. *JAMA Network Open*.

Italian
Jamaican
Japanese
Kenyan
Korean
Kurdish/Kurd
Laotian/Lao
Lebanese
Liberian
Malaysian
Mexican
Mexican American Indian
Middle Eastern/North African
Mnong, Moroccan
Nepalese/Nepali
Nigerian
Pakistani
Palestinian
Polish/Pole
Portuguese
Puerto Rican
Russian
Salvadoran
Scottish
Senegalese/Senegal
Sierra Leonean
Singaporean
Somali, South American
South American Indian
Spanish
Sri Lankan
Syrian
Taiwanese
Thai
Trinidadian
Turkish/Turk
Ugandan
Ukrainian
Uzbekg/Uzbek
Vietnamese
West Indian
West African
Zairean
Guardian Unavailable to Ask
Multi Ethnicity
Patient/Family Declined
Other Ethnicity

© 2024 Martoma RA et al. *JAMA Network Open*.

eAppendix 2. Laboratory Confirmation of Measles Cases

PCN providers notified local health departments of suspected measles cases during this outbreak. On a case-by-case basis, the local public health department applied criteria developed by the CDC and ODH to determine if measles testing was appropriate. When local health departments authorized measles testing, they instructed PCN providers to collect and send nasopharyngeal and serum specimens directly to the ODH for testing.

The CDC, ODH, and local public health departments confirmed a case if laboratory testing of a nasopharyngeal specimen detected measles RNA on reverse-transcription polymerase chain reaction (RT-PCR) or serum was positive for measles-specific immunoglobulin M antibody.(1) Patients who developed a rash within 14 days of MMR vaccination and tested positive for RT-PCR were also tested for measles virus vaccine genotype A (MeVA). A positive MeVA test indicated a vaccine reaction, disqualifying the patient from consideration as a case.

eAppendix 3. Classification of Measles Cases by Date of Rash Onset

We classified cases by date of rash onset. In four cases that documented symptom onset but not rash onset, we estimated the date of rash onset by adding five days to the start of recorded symptoms (fever, cough, congestion, or conjunctivitis). In one case that did not document rash or symptom onset, we estimated the date of rash onset using the “date of onset” notated on laboratory testing forms.

© 2024 Martoma RA et al. *JAMA Network Open*.

eFigure. **Network Diagram of the 2022-2023 Central Ohio Measles Outbreak**

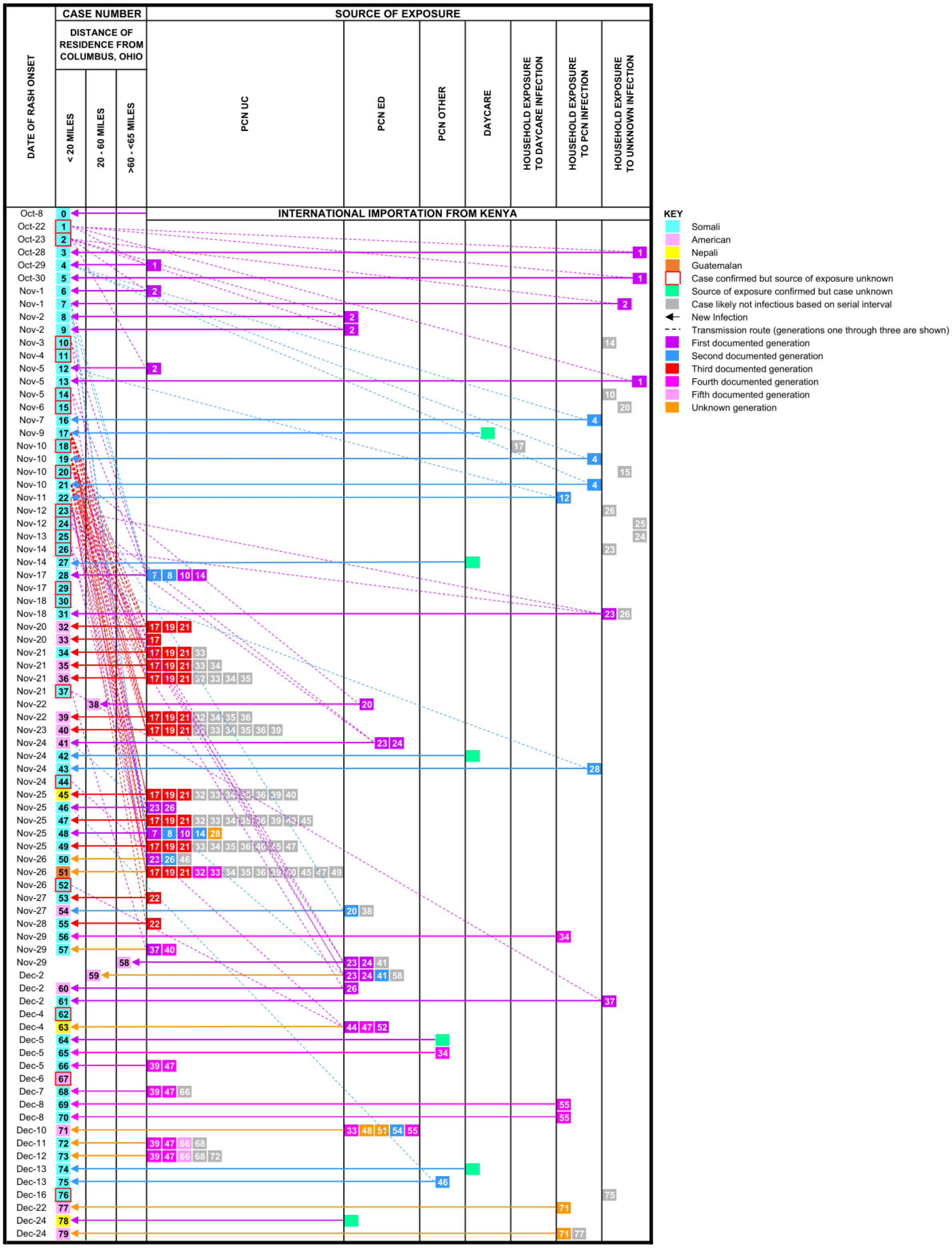

This diagram illustrates the demographic characteristics and transmission pathways of 80 cases identified at a Primary Care Network during the 2022-2023 Central Ohio measles outbreak.

© 2024 Martoma RA et al. *JAMA Network Open*.

eReference.

1. Gastanaduy PA, Redd SB, Clemmons NS, Lee AD, Hickman CJ, Rota PA, et al. Manual for the surveillance of vaccine-preventable diseases. Center for Disease Control and Prevention https://wwwcdcgov/vaccines/pubs/surv-manual/chpt07-measleshtml. 2019.

© 2024 Martoma RA et al. *JAMA Network Open*.
