# Supplementary material for: Epidemiologic Analysis of a Postelimination Measles Outbreak in Central Ohio, 2022–2023
Source: JAMA Netw Open. Author manuscript; Available in PMC 2025 Oct 9. (PMC12509376; doi:10.1001/jamanetworkopen.2024.29696)
Supplement: Supp 2 [file NIHMS2109843-supplement-Supp_2.pdf]

Data Sharing Statement

Martoma. Epidemiologic Analysis of a Postelimination Measles Outbreak in Central Ohio, 2022-2023. *JAMA Netw Open*. Published August 26, 2024.
doi:10.1001/jamanetworkopen.2024.29696

Data
Data available: No

Additional Information
Explanation for why data not available: Due to privacy regulations and the need to protect patient confidentiality, this study does not share identifiable health information (PHI). Data sharing is restricted to ensure compliance with privacy laws and to safeguard the personal and medical information of individuals involved in the study.
